# (*E*)-1-[4-(Methyl­sulfan­yl)phen­yl]-3-phenyl­prop-2-en-1-one

**DOI:** 10.1107/S1600536808017200

**Published:** 2008-06-13

**Authors:** A. Thiruvalluvar, M. Subramanyam, R. J. Butcher, T. Karabasanagouda, A. V. Adhikari

**Affiliations:** a PG Research Department of Physics, Rajah Serfoji Government College (Autonomous), Thanjavur 613 005, Tamil Nadu, India; b Department of Chemistry, Howard University, 525 College Street NW, Washington, DC 20059, USA; c Department of Chemistry, National Institute of Technology Karnataka, Surathkal, Srinivasnagar 575 025, India

## Abstract

In the title mol­ecule, C_16_H_14_OS, the dihedral angle between the phenyl and benzene rings is 3.81 (15)°. The H atoms of the central enone group are *trans*. The propenone unit makes dihedral angles of 11.73 (18) and 11.62 (17)° with the benzene and phenyl rings, respectively. The crystal structure is stabilized by weak C—H⋯O and C—H⋯π inter­actions.

## Related literature

For related crystal structures, see Sathiya Moorthi *et al.* (2005 ▶); Moorthi *et al.* (2005 ▶); Thiruvalluvar *et al.* (2007*a* ▶,*b* ▶).
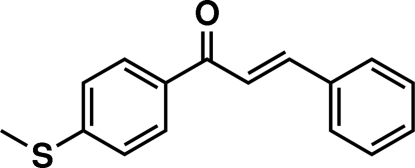


## Experimental

### 

#### Crystal data

C_16_H_14_OS
*M* *_r_* = 254.34
Orthorhombic, 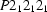

*a* = 5.6106 (4) Å
*b* = 7.6239 (7) Å
*c* = 30.477 (2) Å
*V* = 1303.64 (17) Å^3^
*Z* = 4
Mo *K*α radiation
μ = 0.23 mm^−1^
*T* = 200 (2) K
0.49 × 0.18 × 0.15 mm

#### Data collection

Oxford Diffraction Gemini R diffractometer
Absorption correction: multi-scan (*CrysAlis RED*; Oxford Diffraction, 2007 ▶) *T*_min_ = 0.849, *T*_max_ = 1.000 (expected range = 0.820–0.966)
8924 measured reflections
4060 independent reflections
3180 reflections with *I* > 2σ(*I*)
*R*_int_ = 0.069

#### Refinement

*R*[*F* ^2^ > 2σ(*F* ^2^)] = 0.077
*wR*(*F* ^2^) = 0.181
*S* = 1.11
4060 reflections
163 parameters
H-atom parameters constrained
Δρ_max_ = 0.69 e Å^−3^
Δρ_min_ = −0.38 e Å^−3^
Absolute structure: Flack (1983 ▶), 1314 Friedel pairs
Flack parameter: 0.11 (16)

### 

Data collection: *CrysAlis CCD* (Oxford Diffraction, 2007 ▶); cell refinement: *CrysAlis CCD*; data reduction: *CrysAlis RED* (Oxford Diffraction, 2007 ▶); program(s) used to solve structure: *SHELXS97* (Sheldrick, 2008 ▶); program(s) used to refine structure: *SHELXL97* (Sheldrick, 2008 ▶); molecular graphics: *ORTEP-3* (Farrugia, 1997 ▶); software used to prepare material for publication: *PLATON* (Spek, 2003 ▶).

## Supplementary Material

Crystal structure: contains datablocks global, I. DOI: 10.1107/S1600536808017200/wn2267sup1.cif

Structure factors: contains datablocks I. DOI: 10.1107/S1600536808017200/wn2267Isup2.hkl

Additional supplementary materials:  crystallographic information; 3D view; checkCIF report

## Figures and Tables

**Table 1:** Hydrogen-bond geometry (Å, °)

*D*—H⋯*A*	*D*—H	H⋯*A*	*D*⋯*A*	*D*—H⋯*A*
C3—H3⋯O1	0.95	2.42	2.781 (4)	102
C12—H12⋯*Cg*1^i^	0.95	2.99	3.704 (3)	133
C15—H15⋯*Cg*1^ii^	0.95	2.89	3.488 (3)	122
C25—H25⋯*Cg*2^iii^	0.95	2.90	3.562 (3)	127

Symmetry codes: (i) 
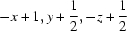
; (ii) 
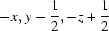
; (iii) 
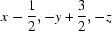
. *Cg*1 and *Cg*2 are the centroids of the C11–C16 and C21–C26 rings, respectively.

**Fig. 1.**
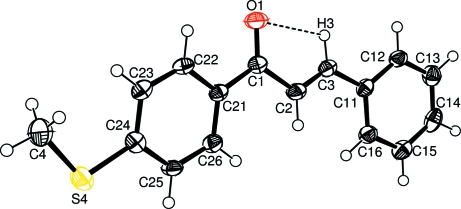
The molecular structure of the title compound, showing the atom-numbering scheme and displacement ellipsoids drawn at the 50% probability level. Hydrogen atoms are represented by spheres of arbitrary radius.

## supplementary crystallographic information

### Comment

The title compound (Fig. 1) has been analysed as part of our crystallographic studies on chalcones (Thiruvalluvar *et al.* 2007a,b). The dihedral angle between the phenyl and benzene rings is 3.81 (15)°; the two rings are essentially coplanar. The H atoms of the central enone group are *trans*. The propenone unit makes dihedral angles of 11.73 (18)° and 11.62 (17)° with the benzene and phenyl rings, respectively.

The crystal structure is stabilized by a weak C3—H3···O1 intramolecular interaction. Furthermore, C12—H12···π, C15—H15···π and C25—H25···π interactions are also found. In similar structures, such as 1-(4-aminophenyl)-3-(3-bromophenyl)-prop-2-en-1-one (Sathiya Moorthi, *et al.* 2005) and 1-(4-bromophenyl)-3-(3-hydroxy phenyl)prop-2-en-1-one (Moorthi, *et al.* 2005), the dihedral angles between the two rings are 9.6 (1)° and 10.2 (2)°, respectively.

### Experimental

Benzaldehyde (2.12 g, 0.02 mol) in ethanol (22 ml) was mixed with 4'-(methylthio)acetophenone (3.32 g, 0.02 mol) in 40 ml ethanol and the mixture was treated with 10 ml of 10% sodium hydroxide solution at 283 K and stirred at 303–305 K for 8 h. The precipitate obtained was filtered, washed with chilled ethanol and dried. Pale yellow rods of the title compound were grown from toluene by slow evaporation. The yield of the isolated product was 3.4 g (70%).

### Refinement

H atoms were positioned geometrically and allowed to ride on their parent atoms, with C—H = 0.95 and 0.98 Å for Csp^2^ and methyl C, respectively; *U*_iso_(H) = x*U*_eq_(C), where x = 1.5 for methyl H and 1.2 for all other H. There was some minor non-merohedral twinning, resulting in *F_o_*^2^ being consistently larger than Fc^2^. The 37 most affected reflections were omitted.

### Crystal data

**Table:**

C_16_H_14_OS	*D*_x_ = 1.296 Mg m^−^^3^
*M**_r_* = 254.34	Melting point: 396(1) K
Orthorhombic, *P*2_1_2_1_2_1_	Mo *K*α radiation λ = 0.71073 Å
Hall symbol: P 2ac 2ab	Cell parameters from 3999 reflections
*a* = 5.6106 (4) Å	θ = 4.7–32.4º
*b* = 7.6239 (7) Å	µ = 0.23 mm^−^^1^
*c* = 30.477 (2) Å	*T* = 200 (2) K
*V* = 1303.64 (17) Å^3^	Needle, colourless
*Z* = 4	0.49 × 0.18 × 0.15 mm
*F*_000_ = 536	

### Data collection

**Table:**

Oxford Diffraction Gemini diffractometer	4060 independent reflections
Radiation source: fine-focus sealed tube	3180 reflections with *I* > 2σ(*I*)
Monochromator: graphite	*R*_int_ = 0.069
Detector resolution: 10.5081 pixels mm^-1^	θ_max_ = 32.5º
*T* = 200(2) K	θ_min_ = 4.7º
φ and ω scans	*h* = −6→8
Absorption correction: multi-scan(CrysAlis RED; Oxford Diffraction, 2007)	*k* = −11→10
*T*_min_ = 0.849, *T*_max_ = 1.000	*l* = −45→38
8924 measured reflections	

### Refinement

**Table:**

Refinement on *F*^2^	Hydrogen site location: inferred from neighbouring sites
Least-squares matrix: full	H-atom parameters constrained
*R*[*F*^2^ > 2σ(*F*^2^)] = 0.077	*w* = 1/[σ^2^(*F*_o_^2^) + (0.0605*P*)^2^ + 1.4543*P*] where *P* = (*F*_o_^2^ + 2*F*_c_^2^)/3
*wR*(*F*^2^) = 0.182	(Δ/σ)_max_ < 0.001
*S* = 1.11	Δρ_max_ = 0.69 e Å^−^^3^
4060 reflections	Δρ_min_ = −0.38 e Å^−^^3^
163 parameters	Extinction correction: none
Primary atom site location: structure-invariant direct methods	Absolute structure: Flack (1983), 1314 Friedel pairs
Secondary atom site location: difference Fourier map	Flack parameter: 0.11 (16)

### Special details

**Table:**

Geometry. Bond distances, angles *etc*. have been calculated using the rounded fractional coordinates. All su's are estimated from the variances of the (full) variance-covariance matrix. The cell e.s.d.'s are taken into account in the estimation of distances, angles and torsion angles
Refinement. Refinement of *F*^2^ against ALL reflections. The weighted *R*-factor *wR* and goodness of fit *S* are based on *F*^2^, conventional *R*-factors *R* are based on *F*, with *F* set to zero for negative *F*^2^. The threshold expression of *F*^2^ > σ(*F*^2^) is used only for calculating *R*-factors(gt) *etc*. and is not relevant to the choice of reflections for refinement. *R*-factors based on *F*^2^ are statistically about twice as large as those based on *F*, and *R*- factors based on ALL data will be even larger.

### Fractional atomic coordinates and isotropic or equivalent isotropic displacement parameters (Å^2^)

**Table:**

	*x*	*y*	*z*	*U*_iso_*/*U*_eq_	
S4	0.48075 (18)	0.98501 (13)	−0.09063 (3)	0.0418 (3)	
O1	0.8645 (4)	1.0222 (4)	0.11787 (7)	0.0411 (8)	
C1	0.6679 (5)	0.9800 (4)	0.10480 (9)	0.0284 (8)	
C2	0.4803 (6)	0.9187 (4)	0.13565 (9)	0.0300 (8)	
C3	0.5318 (6)	0.8989 (4)	0.17807 (9)	0.0273 (8)	
C4	0.7257 (8)	1.0997 (6)	−0.11513 (11)	0.0481 (13)	
C11	0.3725 (6)	0.8391 (4)	0.21292 (9)	0.0269 (8)	
C12	0.4425 (5)	0.8603 (4)	0.25687 (9)	0.0284 (8)	
C13	0.2984 (7)	0.8019 (5)	0.29078 (10)	0.0349 (10)	
C14	0.0835 (7)	0.7219 (5)	0.28169 (11)	0.0374 (10)	
C15	0.0098 (7)	0.6998 (4)	0.23848 (10)	0.0335 (9)	
C16	0.1525 (6)	0.7588 (4)	0.20435 (10)	0.0306 (9)	
C21	0.6093 (5)	0.9893 (4)	0.05707 (9)	0.0239 (7)	
C22	0.7753 (5)	1.0701 (4)	0.02917 (10)	0.0285 (8)	
C23	0.7412 (6)	1.0748 (4)	−0.01573 (10)	0.0296 (8)	
C24	0.5389 (6)	0.9955 (4)	−0.03416 (9)	0.0281 (8)	
C25	0.3704 (5)	0.9185 (4)	−0.00654 (10)	0.0293 (8)	
C26	0.4044 (6)	0.9151 (4)	0.03850 (10)	0.0300 (8)	
H2	0.32442	0.89374	0.12518	0.0360*	
H3	0.69018	0.92687	0.18661	0.0328*	
H4A	0.70702	1.09997	−0.14710	0.0720*	
H4B	0.87527	1.04105	−0.10734	0.0720*	
H4C	0.72878	1.22076	−0.10434	0.0720*	
H12	0.59023	0.91523	0.26341	0.0340*	
H13	0.34767	0.81704	0.32036	0.0419*	
H14	−0.01452	0.68175	0.30504	0.0449*	
H15	−0.13808	0.64431	0.23233	0.0402*	
H16	0.10064	0.74459	0.17489	0.0367*	
H22	0.91377	1.12262	0.04138	0.0341*	
H23	0.85442	1.13149	−0.03407	0.0356*	
H25	0.23077	0.86768	−0.01875	0.0351*	
H26	0.28802	0.86225	0.05687	0.0360*	

### Atomic displacement parameters (Å^2^)

**Table:**

	*U*^11^	*U*^22^	*U*^33^	*U*^12^	*U*^13^	*U*^23^
S4	0.0460 (5)	0.0497 (5)	0.0297 (3)	−0.0035 (5)	−0.0048 (3)	−0.0008 (3)
O1	0.0278 (12)	0.0601 (17)	0.0353 (11)	−0.0141 (13)	−0.0040 (9)	0.0017 (11)
C1	0.0259 (14)	0.0295 (15)	0.0297 (12)	−0.0040 (13)	0.0011 (10)	−0.0007 (11)
C2	0.0243 (14)	0.0349 (15)	0.0308 (12)	−0.0088 (13)	0.0007 (11)	0.0004 (11)
C3	0.0215 (14)	0.0254 (13)	0.0350 (13)	−0.0010 (12)	0.0022 (11)	−0.0006 (11)
C4	0.051 (2)	0.059 (3)	0.0342 (16)	−0.005 (2)	0.0029 (15)	0.0084 (17)
C11	0.0248 (14)	0.0252 (14)	0.0307 (13)	0.0026 (12)	0.0026 (11)	0.0010 (11)
C12	0.0218 (15)	0.0295 (15)	0.0338 (14)	0.0040 (11)	−0.0026 (11)	−0.0009 (11)
C13	0.0377 (18)	0.0378 (18)	0.0293 (14)	0.0044 (15)	0.0003 (13)	0.0019 (12)
C14	0.0379 (19)	0.0348 (18)	0.0396 (17)	0.0129 (14)	0.0114 (13)	0.0081 (14)
C15	0.0303 (16)	0.0246 (14)	0.0456 (16)	−0.0027 (14)	0.0050 (14)	0.0008 (11)
C16	0.0262 (15)	0.0323 (16)	0.0332 (14)	0.0015 (13)	−0.0002 (12)	−0.0002 (12)
C21	0.0210 (12)	0.0210 (12)	0.0297 (12)	0.0003 (11)	0.0004 (9)	0.0002 (11)
C22	0.0213 (13)	0.0300 (15)	0.0341 (14)	−0.0069 (12)	0.0002 (11)	0.0004 (12)
C23	0.0255 (14)	0.0295 (15)	0.0339 (14)	−0.0023 (12)	0.0034 (11)	0.0025 (12)
C24	0.0326 (15)	0.0220 (12)	0.0297 (12)	0.0040 (13)	−0.0023 (10)	0.0004 (11)
C25	0.0202 (13)	0.0319 (15)	0.0357 (14)	−0.0070 (12)	−0.0032 (11)	−0.0034 (12)
C26	0.0258 (15)	0.0316 (15)	0.0327 (14)	−0.0035 (13)	0.0022 (11)	0.0001 (12)

### Geometric parameters (Å, °)

**Table:**

S4—C4	1.792 (4)	C24—C25	1.395 (4)
S4—C24	1.754 (3)	C25—C26	1.386 (4)
O1—C1	1.216 (4)	C2—H2	0.9500
C1—C2	1.487 (4)	C3—H3	0.9500
C1—C21	1.493 (4)	C4—H4A	0.9800
C2—C3	1.333 (4)	C4—H4B	0.9800
C3—C11	1.461 (4)	C4—H4C	0.9800
C11—C12	1.405 (4)	C12—H12	0.9500
C11—C16	1.402 (5)	C13—H13	0.9500
C12—C13	1.386 (4)	C14—H14	0.9500
C13—C14	1.379 (5)	C15—H15	0.9500
C14—C15	1.391 (5)	C16—H16	0.9500
C15—C16	1.388 (5)	C22—H22	0.9500
C21—C22	1.404 (4)	C23—H23	0.9500
C21—C26	1.401 (4)	C25—H25	0.9500
C22—C23	1.382 (4)	C26—H26	0.9500
C23—C24	1.403 (5)		
			
S4···H16^i^	3.1800	H2···C26	2.6800
O1···H2^ii^	2.7700	H2···H16	2.2700
O1···H3	2.4200	H2···H26	2.1100
O1···H22	2.4700	H3···O1	2.4200
O1···H4C^iii^	2.8600	H3···C15^ii^	2.9500
O1···H14^iv^	2.7800	H3···C16^ii^	2.9400
C3···C13^iv^	3.354 (5)	H3···H12	2.4100
C3···C14^iv^	3.497 (5)	H3···C13^iv^	2.9400
C3···C15^ii^	3.590 (5)	H3···C14^iv^	2.7600
C12···C15^ii^	3.456 (5)	H4A···C14^x^	3.0300
C13···C3^v^	3.354 (5)	H4A···H14^x^	2.4600
C14···C3^v^	3.497 (5)	H4B···C23	2.9000
C15···C12^vi^	3.456 (5)	H4B···H23	2.3400
C15···C3^vi^	3.590 (5)	H4C···C23	2.9200
C2···H16	2.7800	H4C···H23	2.3600
C2···H26	2.6700	H4C···O1^ix^	2.8600
C4···H23	2.5900	H12···C15^ii^	2.9700
C12···H15^vii^	2.7800	H12···H3	2.4100
C12···H15^ii^	2.9700	H14···H4A^viii^	2.4600
C13···H15^vii^	2.8500	H14···O1^v^	2.7800
C13···H3^v^	2.9400	H15···C12^vi^	2.9700
C14···H3^v^	2.7600	H15···C12^xi^	2.7800
C14···H4A^viii^	3.0300	H15···C13^xi^	2.8500
C15···H3^vi^	2.9500	H16···C2	2.7800
C15···H12^vi^	2.9700	H16···H2	2.2700
C16···H2	2.7900	H16···S4^xii^	3.1800
C16···H3^vi^	2.9400	H22···O1	2.4700
C21···H25^i^	3.0400	H22···C23^iii^	3.0500
C23···H4B	2.9000	H22···C24^iii^	3.0000
C23···H4C	2.9200	H23···C4	2.5900
C23···H22^ix^	3.0500	H23···H4B	2.3400
C24···H22^ix^	3.0000	H23···H4C	2.3600
C25···H25^i^	3.0700	H25···C21^xii^	3.0400
C26···H2	2.6800	H25···C25^xii^	3.0700
C26···H25^i^	2.8900	H25···C26^xii^	2.8900
H2···O1^vi^	2.7700	H26···C2	2.6700
H2···C16	2.7900	H26···H2	2.1100
			
C4—S4—C24	104.13 (16)	C2—C3—H3	116.00
O1—C1—C2	121.2 (3)	C11—C3—H3	116.00
O1—C1—C21	120.4 (3)	S4—C4—H4A	109.00
C2—C1—C21	118.4 (2)	S4—C4—H4B	109.00
C1—C2—C3	119.7 (3)	S4—C4—H4C	109.00
C2—C3—C11	127.4 (3)	H4A—C4—H4B	109.00
C3—C11—C12	119.1 (3)	H4A—C4—H4C	109.00
C3—C11—C16	122.6 (3)	H4B—C4—H4C	109.00
C12—C11—C16	118.3 (3)	C11—C12—H12	120.00
C11—C12—C13	120.7 (3)	C13—C12—H12	120.00
C12—C13—C14	120.2 (3)	C12—C13—H13	120.00
C13—C14—C15	120.3 (3)	C14—C13—H13	120.00
C14—C15—C16	119.9 (3)	C13—C14—H14	120.00
C11—C16—C15	120.7 (3)	C15—C14—H14	120.00
C1—C21—C22	117.7 (3)	C14—C15—H15	120.00
C1—C21—C26	123.7 (3)	C16—C15—H15	120.00
C22—C21—C26	118.5 (3)	C11—C16—H16	120.00
C21—C22—C23	121.3 (3)	C15—C16—H16	120.00
C22—C23—C24	119.8 (3)	C21—C22—H22	119.00
S4—C24—C23	124.3 (2)	C23—C22—H22	119.00
S4—C24—C25	116.5 (2)	C22—C23—H23	120.00
C23—C24—C25	119.2 (3)	C24—C23—H23	120.00
C24—C25—C26	120.8 (3)	C24—C25—H25	120.00
C21—C26—C25	120.4 (3)	C26—C25—H25	120.00
C1—C2—H2	120.00	C21—C26—H26	120.00
C3—C2—H2	120.00	C25—C26—H26	120.00
			
C4—S4—C24—C23	−1.6 (3)	C11—C12—C13—C14	0.0 (5)
C4—S4—C24—C25	178.6 (3)	C12—C13—C14—C15	−0.2 (6)
O1—C1—C2—C3	−4.2 (5)	C13—C14—C15—C16	−0.2 (5)
C21—C1—C2—C3	176.0 (3)	C14—C15—C16—C11	0.7 (5)
O1—C1—C21—C22	−8.6 (4)	C1—C21—C22—C23	176.1 (3)
O1—C1—C21—C26	168.4 (3)	C26—C21—C22—C23	−1.0 (5)
C2—C1—C21—C22	171.3 (3)	C1—C21—C26—C25	−175.5 (3)
C2—C1—C21—C26	−11.8 (4)	C22—C21—C26—C25	1.4 (5)
C1—C2—C3—C11	−179.5 (3)	C21—C22—C23—C24	−0.9 (5)
C2—C3—C11—C12	−167.1 (3)	C22—C23—C24—S4	−177.5 (2)
C2—C3—C11—C16	13.8 (5)	C22—C23—C24—C25	2.4 (5)
C3—C11—C12—C13	−178.7 (3)	S4—C24—C25—C26	177.9 (2)
C16—C11—C12—C13	0.5 (5)	C23—C24—C25—C26	−1.9 (5)
C3—C11—C16—C15	178.3 (3)	C24—C25—C26—C21	0.0 (5)
C12—C11—C16—C15	−0.9 (5)		

Symmetry codes: (i) *x*+1/2, −*y*+3/2, −*z*; (ii) *x*+1, *y*, *z*; (iii) *x*+1/2, −*y*+5/2, −*z*; (iv) −*x*+1, *y*+1/2, −*z*+1/2; (v) −*x*+1, *y*−1/2, −*z*+1/2; (vi) *x*−1, *y*, *z*; (vii) −*x*, *y*+1/2, −*z*+1/2; (viii) −*x*+1/2, −*y*+2, *z*+1/2; (ix) *x*−1/2, −*y*+5/2, −*z*; (x) −*x*+1/2, −*y*+2, *z*−1/2; (xi) −*x*, *y*−1/2, −*z*+1/2; (xii) *x*−1/2, −*y*+3/2, −*z*.

### Hydrogen-bond geometry (Å, °)

**Table:**

*D*—H···*A*	*D*—H	H···*A*	*D*···*A*	*D*—H···*A*
C3—H3···O1	0.95	2.42	2.781 (4)	102
C12—H12···Cg1^iv^	0.95	2.99	3.704 (3)	133
C15—H15···Cg1^xi^	0.95	2.89	3.488 (3)	122
C25—H25···Cg2^xii^	0.95	2.90	3.562 (3)	127

Symmetry codes: (iv) −*x*+1, *y*+1/2, −*z*+1/2; (xi) −*x*, *y*−1/2, −*z*+1/2; (xii) *x*−1/2, −*y*+3/2, −*z*.
